# Protein release through nonlethal oncotic pores as an alternative nonclassical secretory pathway

**DOI:** 10.1186/1471-2121-12-46

**Published:** 2011-10-18

**Authors:** William J Chirico

**Affiliations:** 1 Department of Cell Biology and Molecular & Cellular Biology Program, State University of New York Downstate Medical Center, 450 Clarkson Ave., Box 5, Brooklyn, NY 11203, USA

## Abstract

**Background:**

Nonclassical (unconventional) protein secretion is thought to represent the primary secretion mechanism for several cytosolic proteins, such as HIV-Tat, galectin 1, interleukin-1β, and several proteins that shuttle between the nucleus and cytosol, such as fibroblast growth factor 1 (FGF1), FGF2, and nucleolin. Four nonclassical secretory pathways have been described including direct transport (presumably through transporters in the plasma membrane), secretion via exosomes, lysosomal secretion, and blebbing. The purpose of this study was to gain mechanistic insight into nonclassical protein secretion using phosphoglycerate kinase 1 (PGK1), a previously identified nonclassical secretory protein, as a reporter protein.

**Results:**

Upon shifting HeLa cells into serum-free media PGK1 was released as a free soluble protein without cell loss. Release occurred in two phases: a rapid early phase and a slow late phase. Using a repertory of inhibitors, PGK1 release was shown not to rely on the classical secretory pathway. However, components of the cytoskeleton partially contributed to its release. Significantly, the presence of serum or bovine serum albumin in the media inhibited PGK1 release.

**Conclusions:**

These results are consistent with a novel model of protein release termed oncotic release, in which a change in the colloidal osmotic pressure (oncotic pressure) upon serum withdrawal creates nonlethal oncotic pores in the plasma membrane through which PGK1 - and likely other nearby proteins - are released before the pores are rapidly resealed. These findings identify an alternative mechanism of release for FGF1, HIV-Tat, and galectin 1 whose reported nonclassical secretion is induced by serum withdrawal. Oncotic release may occur in routine cell biological experiments during which cells are washed with serum-free buffers or media and in pathophysiological conditions, such as edema, during which extracellular protein concentrations change.

## Background

Several important proteins, such fibroblast growth factor 1 (FGF1), FGF2, and interleukin-1β (IL-1β) are secreted from cells by alternative pathways collectively termed nonclassical (unconventional) secretory pathways [1]. Nonclassical secretory proteins are not synthesized as precursors with an N-terminal hydrophobic signal sequence, which is common to classical secretory proteins, and they are not glycosylated. They do not use the endoplasmic reticulum and Golgi apparatus as conduits to the cell surface and their secretion is resistant to brefeldin A (BFA), a potent inhibitor of the classical secretory pathway.

Four nonclassical protein secretory pathways have been described (reviewed in [2]). They include 1) direct transport of proteins from the cytosol across the plasma membrane presumably through membrane transporters, 2) lysosomal secretion, 3) export via exosomes derived from multivesicular bodies, and 4) packaging of proteins into plasma membrane vesicles (blebbing). In addition, cytosolic proteins can exit cells damaged by mechanical means, such as scraping and needle puncture [3]. Although FGF1 and FGF2 can be secreted directly through the plasma membrane, the dependence of FGF1, but not FGF2, secretion on heat shock suggests they are secreted by different mechanisms [4]. Some proteins can leave by more than one pathway depending on cell type or experimental conditions. For example, IL-1β can be exported in secretory lysosomes [5], blebs [6], exosomes [7], or directly through the plasma membrane by unknown transporters [8].

In experiments described in this report, an established nonclassical secretory protein, phosphoglycerate kinase 1 (PGK1), was used to gain insight into the mechanism of nonclassical protein secretion. PGK1 is the sixth enzyme in glycolysis catalyzing the conversion of 1,3 bisphosphoglycerate into 3-phosphoglycerate and yielding ATP. Extracellular PGK1 acts as a disulphide reductase in an enzymatic cascade generating angiostatin from plasmin [9]. PGK1 is an abundant cytosolic protein and its biochemical and structural properties are well established rendering it an excellent model protein to study nonclassical protein secretion [10]. In contrast, many nonclassically secreted proteins, such as FGF1 and FGF2, are found in trace amounts in cells necessitating their overexpression for analysis.

PGK1 can be released from a variety of cells including HeLa [9,11]. It is reported here that PGK1 can be rapidly released from HeLa cells by lowering the colloidal osmotic pressure (oncotic pressure) of the media, a procedure routinely used in cell biology when cells are washed with isoosmotic solutions, such as serum-free media or phosphate buffered saline (PBS). PGK1 exits at discreet sites of disrupted plasma membrane (oncotic pores) without catastrophic cell loss. This process is termed oncotic release.

## Results

### PGK1 as reporter for nonclassical secretion

Hogg and coworkers previously demonstrated that PGK1 can be released from a variety of cells lines including HT1080 cells [9]. PGK1 is an excellent model protein to study nonclassical protein secretion because it is abundant and has several hallmarks of nonclassically secreted proteins. An ELISA assay was used to quantify intracellular and extracellular pools of PGK1 [11]. Immunoblots indicated that PGK1 in both pools is very stable with negligible fragmentation ([11], data not shown). HeLa cells were used as a model system, because they are routinely used in the laboratory and the amount of PGK1 released from them [11] and HT1080 cells [9] were comparable.

### Cell density affects PGK1 release

**Figure 1:**
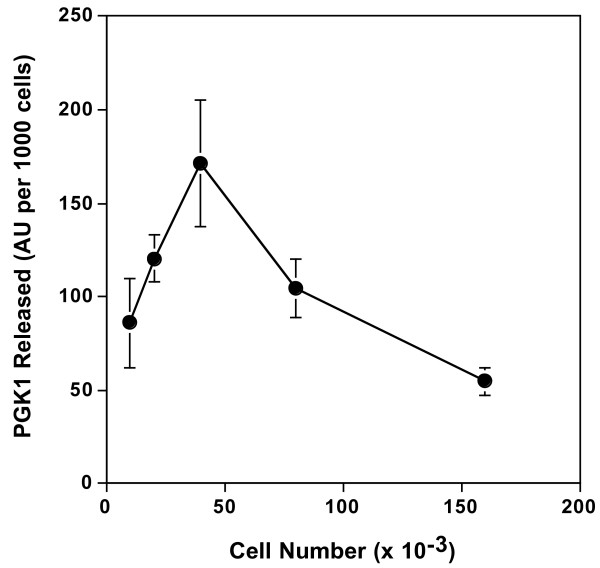
**Effect of cell density on PGK1 release**. HeLa cells were plated at the indicated cell densities in MEM/10% NBS. After 24 h, the cells were washed with serum-free MEM and then incubated with the same media for 2 h. The amount of PGK1 released into the media was determined using an ELISA. Error bars represent the s.d. of the mean (n = 4). AU, arbitrary units.

During the optimization of the PGK1 release assay it was noticed that release was less efficient in more confluent cultures. To determine the effect of cell density on PGK1 release HeLa cells were plated at different densities, allowed to grow for 24 h, and then the amount of PGK1 released into serum-free media during a 2-h incubation was measured. Although PGK1 was released at all cell densities tested, the efficiency of release on a per cell basis was maximal at 40,000 cells/well (Figure 1). This result suggested that cell-cell interactions attenuate PGK1 release and established 40,000 cells/well (21,000 cells/cm^2^) as the optimal cell density for the experiments.

### Time course of PGK1 release

**Figure 2:**
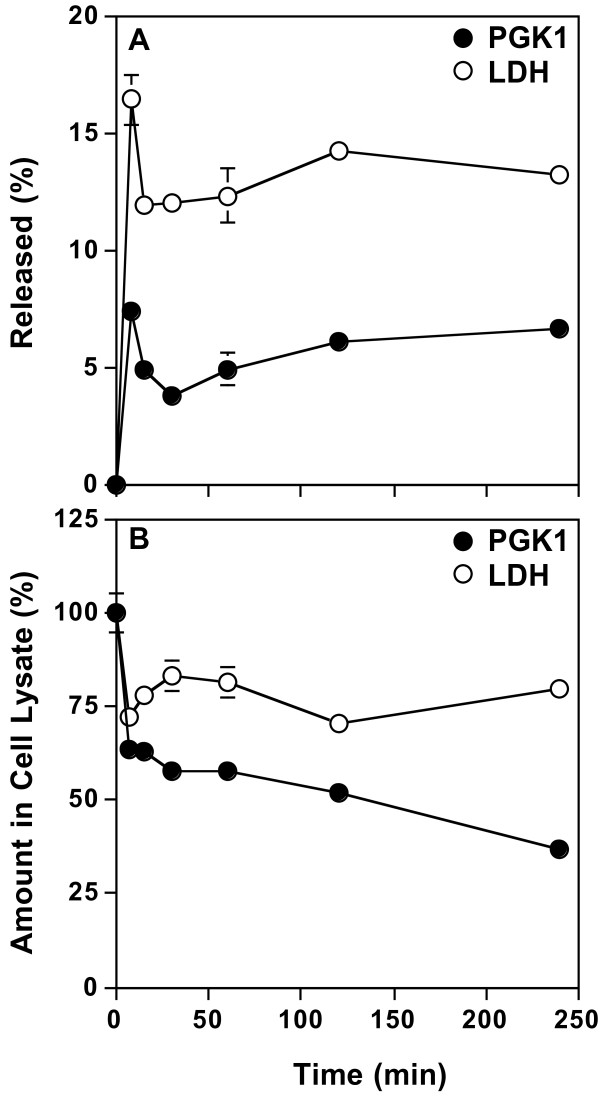
**Time course of PGK1 release**. After HeLa cells were grown in MEM/10% NBS for 24 h, the cells were washed once with serum-free MEM and then incubated in the same media for the indicated times. The amount of PGK1 and LDH released (**A**) and remaining in cell lysates (**B**) was determined and expressed as a percent of the total of each protein in cell lysates at 0 min (untreated cells). Error bars represent the s.d. of the mean (n = 4).

To gain insight into the release process, the time course of PGK1 release from HeLa cells into serum-free medium was measured (Figure 2A). LDH activity was measured to monitor cytotoxicity. Although PGK1 and LDH were released from cells in two phases: a rapid early phase (<30 min) and a slow later phase (30-240 min), the extent of release was different. About 7.5% of the amount of cellular PGK1 at the beginning of the experiment was detected in the media by 7.5 min. By 30 min, the amount of PGK1 in the media decreased to about 4% (P < 0.05). In the slow second phase, the amount of PGK in the media gradually increased from 4% to about 7% (P < 0.05). In contrast, about 16% of the amount of LDH in the cell at the beginning of the experiment was released by 7.5 min and this amount decreased to 12% by 30 min (P < 0.05). The amount of LDH in the media then increased slightly to 13% (P < 0.05) by 240 min. These results suggest that PGK1 and LDH can be released rapidly into serum-free media and that some of the extracellular PGK1 and LDH can be degraded or rendered undetectable to their respective assays.

Interestingly, the amount of PGK detected in the media did not equal the amount of PGK1 lost from the cell (Figure 2B). The amount of cellular PGK1 rapidly decreased to 60% of the original concentration during the first 30 min and then gradually decreased to 40% by 240 min (P < 0.05, Figure 2B). The sudden decrease in the amount of PGK1 in the cell may result from its rapid release, degradation within the cell, or both upon shifting the cells into serum-free media. In contrast, the amount of LDH in the cell decreased to 75% of the original amount by 7.5 min (P < 0.05). By 30 min, the cellular LDH concentration recovered to about 85% (P < 0.05) of the original amount suggesting that some LDH was reabsorbed or replenished by the cell. The concentration of LDH in the cell, in contrast to that of PGK1, was relatively constant at about 80% of the original amount between 30 and 240 min.

**Figure 3:**
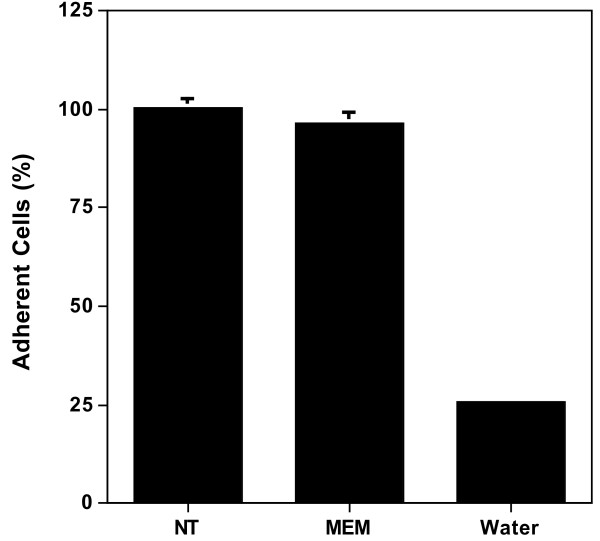
**Cell loss does not accompany serum-withdrawal**. HeLa cells were grown for 24 h in MEM/10% NCS and then either left untreated (NT) or washed once with serum-free MEM and then incubated in the same media for 1 h (MEM). A parallel set of samples was treated with water (Water). Cell number was assayed using SYTOX Green and expressed as the percent remaining relative to the untreated sample. Error bars represent the s.d. of the mean (n = 4).

To test the possibility that PGK1 and LDH release accompanied catastrophic cell loss, the number of cells (based on DNA content) remaining after transferring them into serum-free media was measured (Figure 3). The number of cells remained essentially unchanged after 1 h, a point at which the cellular concentration of PGK1 and LDH decreased, respectively, by 40% and 20%. In contrast, 75% of cells were lost after treating them with water presumably due to hypoosmotic swelling and lysis. This result supports the idea that catastrophic cell loss does not accompany PGK1 and LDH release.

### Vesicle-independent release of PGK1

**Figure 4:**
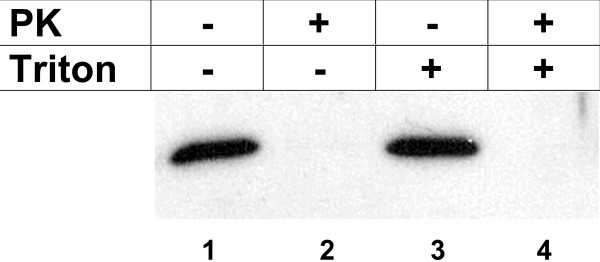
**PGK1 released as a free soluble protein**. Media was collected from HeLa cell cultures 7.5 min after serum withdrawal and then treated with (lanes 2 and 4) or without Proteinase K (PK, lanes 1 and 3) in the presence (lanes 3 and 4) or absence (lanes 1 and 2) of Triton X-100 (Triton) as described in Materials and Methods. Samples were separated on SDS-PAGE gels and then immunoblotted with anti-PGK1 antibodies.

Some nonclassically secreted proteins are packaged into vesicles before or during their release from cells. For example, galectin 3 has been localized in membrane blebs [12] and under certain conditions IL-1β [7] can be found in exosomes. To test the hypothesis that PGK1 is released in vesicles a protease protection assay was performed (Figure 4). In this assay, a protein is protected from exogenously added protease if it is contained within a membrane vesicle. Released PGK1 was completely digested with Proteinase K in the presence (lane 4) or absence (lane 2) of a Triton X-100. Untreated PGK1 was stable in the presence (lane 3) or absence (lane 1) of the detergent. Together these results indicate that PGK1 is released as a free soluble protein and not protected within a vesicle.

### Effect of various inhibitors on PGK1 release

To test the hypothesis that PGK1 is released through a nonclassical secretory pathway its release in the presence of brefeldin A (BFA) or Exo1 was monitored. BFA blocks the classical secretory pathway by inhibiting Arf1, a GTP-binding protein required to initiate bud formation (reviewed in [13]). Although the mechanism by which Exo1 inhibits the classical secretory pathway is not completely understood, it likely interferes with the formation of Arf1-containing complexes involved in ER to Golgi transport [14]. BFA inhibited PGK1 and LDH release by 27 and 20%, respectively (Table 1). In contrast, the secretion of prolactin, which is a protein that passes through the classical secretory pathway, was blocked greater than 90% in the presence of BFA (data not shown, [15]). Interestingly, Exo1 did not significantly affect PGK1 or LDH release (Table 1). Together these results suggest that PGK1 and LDH release are similar and that the classical secretory pathway has a minor role in their release.

**Table 1:** Change in the amount of PGK1 and LDH in media and cell lysates after treating HeLa cells with various reagents

**Reagent**	**Percent change within each compartment relative to vehicle (n = 4)**			
	
	**PGK1**		**LDH**	
	
	**Media**	**Cell**	**Media**	**Cell**
	
BFA	-27 ± 8	NS	-20 ± 10	NS
Exo1	NS	NS	NS	-16 ± 4
Glyburide	-24 ± 11	NS	-20 ± 8	NS
Cytochalasin D	-31 ± 9	10 ± 9	-37 ± 2	6 ± 5
Nocodazole	-39 ± 12	NS	-34 ± 6	NS

NS, no significant change

ATP binding cassette (ABC) transporters have been shown to play a role in the nonclassical secretion of IL-1β [16]. Glyburide, an inhibitor of the ABC1 transporter, was used to probe its contribution, if any, to PGK1 release. Glyburide inhibited PGK1 and LDH release by 24 and 20%, respectively, suggesting that ABC transporters contribute fractionally to their release (Table 1).

Cytochalasin D, an inhibitor of actin polymerization, inhibited PGK1 and LDH release by 31 and 37%, respectively (Table 1). Similarly, nocodazole, an inhibitor of microtubule polymerization, inhibited PGK1 and LDH release by 39 and 34%, respectively (Table 1). Together these results support the idea that PGK1 and LDH are released from cells by a pathway dependent, in part, on the actin and microtubule components of the cytoskeleton.

The effects of other inhibitors of nonclassical secretory pathways were also examined (Additional file 1, Table S1). The inhibitors included methylamine (inhibitor of endosomal and lysosomal function), ouabain (inhibitor of the Na^+^,K^+^-ATPase pump), calcimycin (membrane permeable Ca^2+ ^ionophore), and EGTA (membrane impermeable Ca^2+ ^chelator). In each case, the inhibition of PGK1 release was either negligible or may have resulted from the inhibitor-induced loss of cytosolic PGK1 (Additional file 1, Table S1).

### Serum or BSA blocks PGK1 release

**Figure 5:**
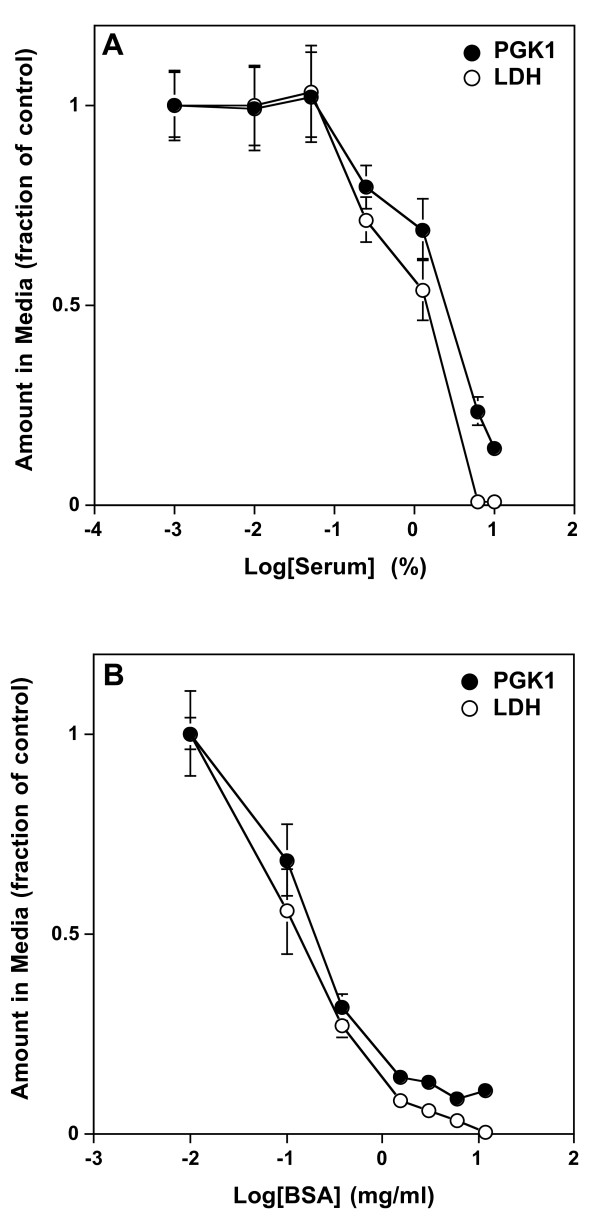
**Serum or BSA blocks release of PGK1 and LDH**. HeLa cells were grown for 24 h in MEM containing 10% serum. After 24 h, media was removed, the cells washed with MEM containing the indicated amounts of serum (**A**) or BSA (**B**), and then incubated with the same media for 7.5 min. The media was collected and the amount of PGK1 and LDH was quantified as described in Materials and Methods. Values are expressed as the ratio of the amount of each protein in the media at each concentration of serum or BSA relative to that in the absence of serum or BSA, respectively. For graphing purposes, the sample lacking serum was defined as 0.001% serum and that lacking BSA as 0.01 mg/ml BSA. Error bars represent the s.d. of the mean (n = 4).

Serum was previously shown to affect the secretion of some nonclassically secreted proteins. For example, serum withdrawal stimulated secretion of FGF1 [17,18] and HIV-1 Tat protein [19], but inhibited secretion of FGF2 [20] and IL-1β [21]. To gain insight into the role of serum in regulating PGK1 release the amount of PGK1 released at different concentrations of serum was measured. The amount of PGK1 released was inversely proportional to serum concentration (Figure 5A). This result indicated that serum attenuates PGK1 release and suggests that the release pathways of FGF1 [17], Tat [19] and PGK1 are, in part, similar.

The findings thus far demonstrate that PGK1 is rapidly released from cells through a process stimulated by serum withdrawal. Two possible explanations for serum regulation of nonclassical secretion were considered. First, the loss of growth factors or cytokines accompanying the shift to serum-free conditions might act as a specific signal for secretion. Second, the loss of total protein from media might act nonspecifically to promote secretion. To distinguish these two possibilities, the effect of different concentrations of BSA on PGK1 release was measured. BSA, like serum, attenuated PGK1 release (Figure 5B). Interestingly the lowest concentration of BSA required to block PGK1 release was 6 mg/ml - about the same concentration as that of total protein in the culture medium (MEM/10% serum). This result supports the idea that changing the total protein concentration in the media influences PGK1 release.

### Confocal microscopy of PGK1 release

**Figure 6:**
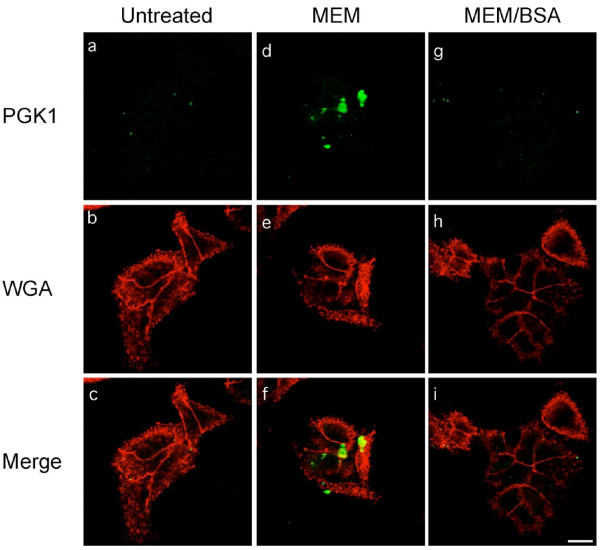
**Confocal images of PGK1 at the cell surface**. HeLa cells were grown in MEM supplemented with 10% NBS. After 24 h, they were either left untreated (**a-c**), washed once with serum-free MEM and then incubated with the same media for 30 min (**d-f**), or washed once with serum-free MEM containing BSA and then incubated with the same media for 30 min (**g-i**). After the incubations the cells were fixed as described in Materials and Methods. Cells were not permeabilized with detergents in order to detect only cell surface proteins. The cell surface was detected using wheat germ agglutinin (WGA, red) and PGK1 (green) was localized using affinity purified rabbit anti-human PGK1 antibody. The bar represents 10 μm (i).

The results suggest that transferring HeLa cells into serum-free media induces the rapid release of PGK1. To identify the site(s) of PGK release the appearance of PGK1 on the cell surface upon shifting cells into serum-free media was monitored using confocal microscopy. To detect PGK1 appearing only on the cell surface the cells were not permeabilized with detergent. Plumes of PGK1 were detected on the surface of cells transferred into serum-free media (Figure 6, panels d-f). In contrast, the amount of PGK1 released from untreated cells (Figure 6, panels a-c) or cells transferred into serum-free media containing BSA (Figure 6, panels g-i) was negligible. These results support the idea that PGK1 is released from intact cells at one or more sites.

### Entry of a membrane impermeable probe under serum-free conditions

SYTOX Green, which is a membrane impermeable fluorescent dye, was used to test the possibility that sites of PGK1 release may permit entry of material into the cell. HeLa cells were incubated for 10 min with SYTOX Green in serum-free or BSA-containing media and the amount of cell-associated fluorescence was measured. About 11-fold more fluorescence was associated with cells incubated with serum-free media than with BSA-containing media (Additional file 2, Figure S1). This result is consistent with the idea that transient sites on the plasma membrane open in response to the acute decrease in extracellular protein concentration allowing cellular components, such as PGK1, to escape and other reagents, such as SYTOX Green, to enter.

## Discussion

In this study PGK1 was used to investigate mechanisms of nonclassical protein secretion. At least 4 models of nonclassical secretion have been proposed including 1) direct passage through a membrane transporter, 2) lysosomal secretion, 3) secretion in exosomes, and 4) blebbing (reviewed in [2]). In addition, mechanically wounding the plasma membrane by scraping can release cytosolic proteins, such as FGF2 [3].

The results presented herein lead to a working model of protein release in which nonlethal oncotic pores or microruptures are formed in the plasma membrane in response to the change in colloidal osmotic pressure upon serum withdrawal allowing the release of PGK1 (and other proteins, for example, LDH) before the pores are rapidly resealed.

Central to the working model of protein release through oncotic pores are the findings that PGK1 release was induced by serum withdrawal and that BSA prevented it. This suggests that its release is triggered by the change in colloidal osmotic pressure (oncotic pressure) instead of a specific factor present in serum. Changes in osmolarity of cell culture media have been demonstrated to alter cell morphology (reviewed in [22]). For example, hyperosmolar solutions shrink cells and hypoosmolar solutions swell cells. The possibility that serum was affecting the osmolarity of the media was excluded by the finding that MEM with or without 10% newborn calf serum had an osmolarity of 320 ± 10 mOsm. Protein solutions, however, have a colligative property that can create a potential energy difference across a semipermeable membrane, such as the cell membrane [23]. This energy difference is termed oncotic pressure or colloidal osmotic pressure [24].

In addition to PGK1, several nonclassically secreted proteins, such as FGF1 [17,18,25,26], S100A13 [17], Tat [19], and galectin 1 [27], are released from cells upon serum withdrawal. A mutated version of rhodanese, which lacked its mitochondrial targeting sequence, was rapidly released from HEK293 cells upon serum withdrawal without any appreciable cell death [28]. Thus, withdrawing or lowering the concentration of serum in media can induce the release of certain proteins by incompletely understood mechanisms.

On the other hand, nonclassical secretory proteins, such as L-29 lectin (galectin-3) [29] and FGF2, under certain conditions [30], can be secreted in the presence of serum. An early report [31] showed FGF2 was released from endothelial cells into serum-free conditioned media while another [20] showed FGF2 secretion from NIH 3T3 cells required serum and serum-free conditions blocked its secretion. Raising the concentration of fetal bovine serum increased the secretion of FGF2 from AIDS-KS cells [32]. Brooks et al. [33] showed that FGF2 can be released from bovine retinal endothelial cells in the presence or absence of serum. Release correlated with cell damage but only after prolonged incubation ranging from 24 to 96 h [33]. These disparate findings likely represent the ability of FGF2 to exit cells by different routes under different experimental conditions.

The above working model of protein release through oncotic pores is consistent with the time course of PGK1 release, cell viability during release, and the effect of cell density on release. The rapid release of PGK1 during the first 30 min may reflect the formation of the pores/microruptures, whereas the attenuation of release may reflect the resealing of the membrane. Catastrophic cell loss did not accompany the release of PGK1 during the formation and subsequent resealing of the plasma membrane. PGK1 release was more efficient at low cell density suggesting that fewer intercellular contacts or more exposed cell surface promote oncotic release.

A variety of inhibitors of classical and nonclassical secretion was used to gain insight into the mechanism of PGK1 release. Resistance of a protein's secretion to inhibitors of the classical secretory pathway, such as BFA, is the hallmark of nonclassical protein secretion [1]. The effect of BFA and Exo1 on PGK1 release was complex - BFA inhibited release by 27%, whereas Exo1 had no significant effect. A comparison of the mechanism of inhibition of BFA and Exo1 suggests that Exo1's effects may be more localized to the Golgi apparatus, whereas those of BFA may be pleiotropic [14,34,35]. For example, BFA, but not Exo1, induces tubulation and collapse of the trans Golgi network and endosomes [14]. And, BFA promotes the release of broader spectrum of proteins from the Golgi than does Exo1 [14,34]. Finally, BFA, but not Exo1, induces the inhibition of CtBP/Bars50, which catalyzes the transfer of palmitate from palmitoyl CoA to lysophosphatidic acid and regulates fission of vesicles from the TGN and plasma membrane [35]. Perhaps inhibiting CtBP/Bars50 influences plasma membrane integrity thereby limiting PGK1 and LDH release during oncotic challenge.

The mechanism by which ABC transporters promote nonclassical protein secretion remains unknown, however, others have suggested they directly transport proteins through their channels or indirectly affect transport during "flipping" of phosphatidylserine [16,36-38]. The partial inhibition of PGK1 release by glyburide raises the possibility that the ion transport activity of some ABC transporters contributes to oncotic damage by allowing cells to swell and oncotic pores/microruptures to form during serum-withdrawal. Glyburide may inhibit PGK1 release by reducing ABC transporter-dependent swelling. Indeed, glyburide has been used to reduce swelling in cerebral edema by inhibiting a nonselective cation channel [39].

Cytochalasin D and nocodazole inhibited PGK1 and LDH release by 30-40% (Table 1) suggesting their release, in part, relies on actin and microtubules. The actin cytoskeleton has been shown to contribute to the nonclassical secretion of FGF1 [17,26] and sphingosine kinase [40]. The microtubule network contributes to galectin 3 nonclassical secretion [41]. Walsh et al. [42] showed that PGK1, LDH and other proteins associate with microtubules. Thus it is possible that the cytoskeleton aids the trafficking or localization of some nonclassically secreted proteins and PGK1 to the plasma membrane and oncotic pores.

The above working model of protein release through oncotic pores accommodates features of nonclassically secreted proteins not addressed directly in this study, such as conformation, charge, and diffusion-limited release. Passage through a large oncotic pore/microrupture would obviate the necessity of protein unfolding during transport. Indeed, neither FGF1 [43] nor FGF2 [44] unfold during secretion. The model offers an explanation for the observation that many, but not all, nonclassically secreted proteins have basic isoelectric points. For example, galectin-3, thioredoxin, FGF2, LDH-A, and PGK1 have pIs of 9.0, 8.9, 9.2, 8.4 and 8.3, respectively. FGF1's pI is 5.2, but its secretion is dependent on its interaction with S100A13 [45], which has a pI of 8.35. A protein's basic isoelectric point may facilitate its interaction with the negatively charged phospholipids of the inner leaflet on the plasma membrane before and during passage through oncotic pores. In addition, the oncotic potential generated by negatively charged serum proteins, such as albumin (pI of 4.7), might draw positively charged cytosolic proteins towards the membrane. Lastly, secretion of some nonclassically secreted proteins, such as FGF2, either by mechanical injury to the cell membrane [46] or by other routes [2] is diffusion controlled. Passage through an oncotic pore/microrupture would likely be rapid.

Although PGK1 can be released through oncotic pores, the possibility remains that it can be released through other routes depending on conditions or cell type. For example, IL-1β can leave cells by plasma membrane shedding, in exosomes derived from multivesicular bodies, or through lysosomal secretion [47]. And FGF2 can be released in vesicles shed from cells [30] or directly through the plasma membrane [48,49].

Release of proteins through oncotic pores and subsequent membrane repair may occur in pathophysiological conditions such as edema, ischemia, and inflammation during which oncotic pressure can change. The presence of PGK1 in serum may serve as an early indicator of plasma membrane damage. In experimental cell biology, cytosolic proteins may escape through cell membranes damaged by mechanical scraping [3] and, as demonstrated herein, through oncotic pores when cells or tissues are gently washed with laboratory buffers, such as PBS, or with isoosmotic serum-free media.

## Conclusions

Oncotic release was identified as novel nonclassical secretory pathway in which cytosolic proteins exit cells through transient nonlethal oncotic pores in the plasma membrane that form in response to the change in colloidal osmotic pressure upon serum withdrawal.

## Methods

### Materials

Minimal Essential Medium (MEM), newborn calf serum (NCS), and glutamine were obtained from Invitrogen. BFA was obtained from Epicentre Technologies. Calcimycin (A23187), cytochalasin D, EGTA, Exo1, glyburide, methylamine, nocodazole, ouabain, and proteinase K were obtained from Sigma. Bovine serum albumin (BSA) (Fraction V) was obtained from Roche. Wheat germ agglutinin (WGA), Alexa Fluor 488 goat anti-rabbit IgG, and SYTOX Green were obtained from Molecular Probes. AffiniPure Goat-Rabbit IgG (Fc Fragment Specific), Biotin-SP-Conjugated AffiniPure Donkey Anti-Chicken IgY, and peroxidase-conjugated streptavidin were purchased from Jackson ImmunoResearch. The preparation of affinity purified rabbit and chicken anti-human PGK1 antibodies was described previously [11].

### Cell Culture

HeLa cells (CCL2) were obtained from ATCC. They were grown in MEM containing 2 mM glutamine and 10% newborn bovine serum (NCS) at 37°C and 5% CO_2_. HeLa cells were used from passages 3 to 10 within the laboratory.

### Buffers

MEM Lysis Buffer was prepared by mixing 20 ml of MEM, 1.1 ml of Triton X-100 (23.4% in PBS), and 1.1 ml of BSA/PIC. BSA/PIC was prepared by dissolving 1 tablet of complete Mini EDTA-free protease inhibitor cocktail (Roche) in 5 ml of 2 mg/ml BSA in PBS.

### PGK1 Release Assay

HeLa cells were plated at a density of 40,000 cells/well of Nunc 4-well multi dish in 0.5 ml of MEM containing with 2 mM glutamine and 10% NCS unless otherwise stated. After 24 h, media was removed, the cells were washed once with 0.5 ml of serum-free MEM containing 2 mM glutamine, and then incubated with the same media for different times. The serum-free media used for washing and capturing released proteins was preincubated overnight at 37°C and 5% CO_2_. During manipulations, culture dishes were placed on a Styrofoam platform to minimize temperature changes and dishes were processed individually. At different times the media was transferred to a 1.5 ml microfuge tube containing 27.8 μl of BSA/PIC on ice. BSA was added to the microfuge tube to reduce potential nonspecific adsorption of released proteins and to stabilize LDH activity. Adherent cells were treated with 555 μl of MEM Lysis Buffer for 45 min at room temperature and then the lysates were kept at 4°C until assayed. The media fraction was centrifuged at 13,000 rpm for 2 min at 4°C to remove any dislodged cells. The upper portion of the supernatant (422 μl) was carefully transferred to a fresh microfuge tube containing 22.2 μl of Triton X-100 (23.4% in PBS). Thus, the final composition of the media fraction was equivalent to that of the MEM Lysis Buffer.

The effect of a variety of inhibitors of the classical and nonclassical secretory pathways on PGK1 release was examined. The minimum effective dose (MED) was determined for each inhibitor (data not shown). HeLa cell cultures were preincubated separately with each inhibitor at its MED for 30 min before starting the release assay described above. The same inhibitor concentration was used during preincubation, wash, and release. The inhibitors used were BFA (10 μM), Exo1 (50 μM), glyburide (100 μM), cytochalasin D (2 μM), nocodazole (10 μM), methylamine (50 mM), ouabain (100 μM), calcimycin (5 μM), and EGTA (5 mM). The change in the amount of PGK1 and LDH in the media and cell lysates was expressed as percent change within each fraction (media or cell lysate) relative to fractions from control cells treated with an equivalent amount of vehicle.

### PGK1 ELISA

The amount of PGK1 in media and cell lysates was quantified using a sandwich ELISA as previously described [11] with the following modifications to increase sensitivity. The ELISA plate was coated with AffiniPure Goat-Rabbit IgG, Fc Fragment Specific. PGK1 was captured using affinity purified rabbit anti-human PGK1 antibody [11]. PGK1 was detected using the sequential application of affinity purified chicken anti-human PGK1 antibody [11], biotin-SP-conjugated AffiniPure donkey anti-chicken IgY, and peroxidase-conjugated streptavidin.

### Lactate Dehydrogenase Assay (LDH)

The activity of LDH in media and cell lysates was measured using the CytoTox 96 Non Radioactive Cytotoxicity Assay (Promega). A standard curve using LDH provided by the manufacturer was done for each experiment in MEM Lysis Buffer. Fractions (media and cell lysates) were kept at 4°C and LDH activity was measured immediately after each experiment.

### Sytox Green Assay

HeLa cells (14,000 cells/well) were incubated in MEM/10% NCS at 37°C and 5% CO_2 _in a 96-well white plates (Nunc). After 24 h, cells were left untreated or washed once with serum-free MEM and then incubated with the same media for 1 h. A parallel set of samples was treated with water instead of serum-free MEM. At the end of the incubation period, the media (or water) was removed and the cells treated with 100 μl of ethanol:acetone (1:1) for 10 min. The ethanol/acetone mixture was removed and the samples were treated with 100 μl of PBS for 10 min. The PBS was removed and the samples incubated with 100 μl of 125 nM Sytox Green in PBS for 30 min. The fluorescence emission was measured using a Victor^3 ^1420 Multicolor Fluorescence Plate Reader (Perkin Elmer).

### Protease Protection Assay

HeLa cells were grown under the standard conditions described above for monitoring PGK1 release. After 24 h, cells were washed once with serum-free MEM and then incubated for 7.5 min in the same media at 37°C and 5% CO_2_. The media was transferred into a 1.5 ml microfuge tube and then centrifuged for 2 min at 4°C. The supernatant was transferred to a fresh tube and stored on ice. Aliquots (120 μl) of the supernatant were treated with 4 μl of either proteinase K (10 mg/ml in 50 mM Tris-HCl, pH 8, 10 mM CaCl_2_) or buffer alone in the presence or absence of a final concentration of 1% Triton X-100. After the samples were incubated for 2 h at 25°C, an aliquot was removed, mixed with an equal volume of 2X SDS-PAGE sample buffer containing DTT, and the mixture boiled for 5 min. Equivalent volumes were separated on 12% SDS-PAGE gels [50] and immunoblotted [50] with rabbit anti-PGK1 antibody.

### Confocal Microscopy

HeLa cells were grown on polylysine coated coverslips in MEM containing 10% NCS. After 24 h, they were washed once with serum-free MEM and then incubated with 0.5 ml of the same media for 30 min at 37°C and 5% CO_2_. Two controls were included in the experiment. In the unperturbed control, a HeLa cell culture was placed on ice, the media was removed by aspiration, and the cells were fixed. In the oncotically matched control, a culture was washed once with serum-free MEM containing 6 mg/ml BSA, and then incubated with the same media for 30 min at 37°C and 5% CO_2_. The serum-free MEM and the serum-free MEM containing BSA used for washing and incubating cells were preincubated at 37°C/5% CO_2 _and removed from the incubator just before use. At the end of the incubation, the culture dishes were placed on ice, the media removed by aspiration, and the cells treated with 0.5 ml of ice-cold fixative for 30 min on ice. The fixative was 0.25% paraformaldehyde, 0.0125% glutaraldehyde, and 250 mM sucrose in MEM, pH 7.5. The components of the fixative were optimized to preserve the integrity of the cell membrane. After fixation, the cells were washed with PBS and then blocked for 1 h with 3% BSA/1% goat serum in PBS. To detect components at the cell surface, the cells were incubated briefly (15 min) with affinity purified rabbit anti-human PGK1 antibody (1/500, [11]) and Alexa Red conjugated WGA (1/500, Molecular Probes) on ice. Alexa Green conjugated anti-rabbit antibody (1/500) was used as a secondary antibody. Antibodies and WGA were diluted with blocking solution. No detergents were used during the preparation of cells for confocal microscopy. Images were acquired using BioRad Radiance 2000 Confocal Laser Scanning Microscope equipped with Plan NEOFLUAR 40X/1.30 Oil DIC objective and LaserSharp 2000 software.

### Statistical Analysis

Statistical significance between two sets of data was determined using an unpaired *t *test. P < 0.05 was considered significant.

## Authors' contributions

WJC conceived the project, designed and executed the experiments, and wrote the manuscript.

## Author's information

WJC is an Associate Professor of Cell Biology and the Director of the Molecular & Cellular Biology Program at the SUNY Downstate Medical Center.

## Supplementary Material

Additional file 1 **Table S1 contains the results of experiments using additional inhibitors of PGK1 release**. Click here for file

Additional file 2 **Figure S1 demonstrates that SYTOX Green can enter HeLa cells under serum-free conditions**. Click here for file
