# Supplementary material for: Protein release through nonlethal oncotic pores as an alternative nonclassical secretory pathway
Source: BMC Cell Biol. 2011 Oct 18;12:46. doi: 10.1186/1471-2121-12-46 (PMC3217904; doi:10.1186/1471-2121-12-46)
Supplement: Additional file 1 — Table S1 contains the results of experiments using additional inhibitors of PGK1 release. [file 1471-2121-12-46-S1.DOC]

**Additional file 1, Table S1**. Change in the amount of PGK1 and LDH in media and cell lysates after treating HeLa cells with various reagents

| Reagent | Percent change within each compartment  relative to vehicle (n=4) | | | |
| --- | --- | --- | --- | --- |
|  | PGK1 | | LDH | |
|  | Media | Cell | Media | Cell |
| Methylamine | -50 ± 6 | -22 ± 4 | -29 ± 7 | NS |
| Ouabain | -40 ± 8 | -31 ± 4 | -23 ± 9 | NS |
| Calcimycin | NS | -11 ± 6 | 10 ± 4 | NS |
| EGTA | -58 ± 5 | -20 ± 4 | 14 ± 10 | -12 ± 2 |

NS, no significant change
