# Supplementary material for: Protein release through nonlethal oncotic pores as an alternative nonclassical secretory pathway
Source: BMC Cell Biol. 2011 Oct 18;12:46. doi: 10.1186/1471-2121-12-46 (PMC3217904; doi:10.1186/1471-2121-12-46)
Supplement: Additional file 2 — Figure S1 demonstrates that SYTOX Green can enter HeLa cells under serum-free conditions. [file 1471-2121-12-46-S2.DOC]

**Additional file 2, Fig. S1. Entry of membrane impermeable probe into HeLa cells under serum-free conditions.**  HeLa cells (14,000 cells/well) were grown in MEM containing 10% NCS. After 24 h, the cells were washed once with 125 nm SYTOX Green in either serum-free MEM or MEM containing 6 mg/ml BSA and then incubated with the same solution for 10 min. The cells were washed, respectively, with PBS or PBS containing 6 mg/ml BSA. Background fluorescence of PBS or the BSA-containing PBS was subtracted from the corresponding samples containing cells. Values are the mean ± SD, N=4, P<0.05.
